# Management of Hepatitis E Virus (HEV) Zoonotic Transmission: Protection of Rabbits against HEV Challenge following Immunization with HEV 239 Vaccine

**DOI:** 10.1371/journal.pone.0087600

**Published:** 2014-01-30

**Authors:** Peng Liu, Ren jie Du, Ling Wang, Jian Han, Lin Liu, Yu lin Zhang, Jun ke Xia, Feng min Lu, Hui Zhuang

**Affiliations:** Department of Microbiology, School of Basic Medical Sciences, Peking University, Beijing, China; Cornell University, United States of America

## Abstract

Hepatitis E virus (HEV) constitutes a significant health burden worldwide, with an estimated approximately 33% of the world’s population exposed to the pathogen. The recent licensed HEV 239 vaccine in China showed excellent protective efficacy against HEV of genotypes 1 and 4 in the general population and pregnant women. Because hepatitis E is a zoonosis, it is also necessary to ascertain whether this vaccine can serve to manage animal sources of human HEV infection. To test the efficacy of the HEV 239 vaccine in protecting animal reservoirs of HEV against HEV infection, twelve specific-pathogen-free (SPF) rabbits were divided randomly into two groups of 6 animals and inoculated intramuscularly with HEV 239 and placebo (PBS). All animals were challenged intravenously with swine HEV of genotype 4 or rabbit HEV seven weeks after the initial immunization. The course of infection was monitored for 10 weeks by serum ALT levels, duration of viremia and fecal virus excretion and HEV antibody responses. All rabbits immunized with HEV 239 developed high titers of anti-HEV and no signs of HEV infection were observed throughout the experiment, while rabbits inoculated with PBS developed viral hepatitis following challenge, with liver enzyme elevations, viremia, and fecal virus shedding. Our data indicated that the HEV 239 vaccine is highly immunogenic for rabbits and that it can completely protect rabbits against homologous and heterologous HEV infections. These findings could facilitate the prevention of food-borne sporadic HEV infection in both developing and industrialized countries.

## Introduction

Hepatitis E virus (HEV) constitutes a significant health burden worldwide, especially in regions with poor sanitation including large parts of Asia, Africa and Mexico, where it has proved to be the most or second-most important cause of acute clinical hepatitis [1]. At least four genotypes comprising a single serotype of mammalian HEV exist. Genotypes 1 and 2 exclusively infect humans and are mainly responsible for the large epidemics that have occurred in resource-limited areas where they are transmitted by water-borne and fecal–oral routes usually through contaminated water supplies. Genotypes 3 and 4 are zoonotic, and are mainly associated with sporadic infections and limited foodborne outbreaks in both developing and developed countries [2]. To-date, in addition to human, mammalian HEV strains have been isolated from both domesticated and wild pigs, *sika* deer, mongooses and rabbits, and antibodies to HEV have been detected in a wider range of animal species including cats, dogs, cattle, sheep, goats, horses, macaques, donkeys, rats, and mice [3], [4].

Accumulating lines of evidence indicate that animal reservoirs of HEV serve as important sources of human infection. The demonstration of HEV infection in humans following consumption of undercooked infected meat from wild boar and deer has provided direct proof of zoonotic transmission of HEV genotypes 3 and 4 [5], [6]. Furthermore, swine and rabbit HEVs have been shown experimentally to be capable of crossing the species barrier and infecting non-human primates [7], [8]. In addition, the higher frequency of antibodies to HEV among animal handlers [9], [10] and the close genetic relationship of HEV strains obtained from humans and those from swine in the same geographical regions also support zoonotic transmission is a significant route of the virus spreading [11], [12]. The zoonotic nature of HEV dictates that foodborne infection can possibly be prevented through vaccination of significant animal reservoirs such as pigs and rabbits.

HEV 239 (Hecolin; Xiamen Innovax Biotech, Xiamen, China), which was approved by China’s State Food and Drug Administration (SFDA) in December 2011, is the world’s first commercial vaccine against HEV infection [13]. The results of a Phase III trial in China involving 11,165 healthy men and women aged 16–65 years showed a protective efficacy of 100% and no unexpected side effects in the general population [14] and pregnant women [15]. In the present study, we evaluated the efficacy of the HEV 239 vaccine in protecting rabbits against homologous and heterologous HEV infections, aiming to examine whether HEV 239 could serve to manage HEV transmission from its animal reservoirs.

## Materials and Methods

### Ethics statement

The animal experiments were approved by the Committee of Laboratory Animal Welfare and Ethics, Peking University Health Science Center. The regulations of the review committee of Laboratory Animal Welfare and Ethics and the protocol for the review on Laboratory Animal Welfare and Ethics, Peking University Health Science Center, were followed.

### HEV 239 vaccine

The HEV 239 vaccine (Hecolin; Xiamen Innovax Biotech, Xiamen, China) is a 26 kDa recombinant polypeptide corresponding to amino acid residues 368–606 of the capsid protein of a genotype 1 HEV strain [16]. The vaccine is expressed in *Escherichia coli* (*E. coli*) and vaccine doses contain 30 µg of the purified antigen in 0.5 mL buffered saline adsorbed to 0.8 mg aluminium hydroxide.

### Specific-pathogen-free rabbits

Twelve, 6-week-old, specific-pathogen-free (SPF) New Zealand White rabbits were divided randomly into two groups of 6 animals, to serve as a placebo group and vaccine group, respectively. All rabbits were confirmed negative for anti-HEV antibodies by an enzyme-linked immunosorbent assay (ELISA) and individually housed in a BSL-2 facility.

### Challenge viruses

A genotype 4 strain bjsw1 (pig feces, GU206559) and a rabbit strain of HEV CHN-BJ-R14 (rabbit feces, JX109834) were used as challenge viruses. A suspension [in phosphate-buffered saline (PBS, pH 7.4)] of feces containing the swine or rabbit HEV strain was prepared as a challenge pool. The clarified suspension was subsequently filtered through 0.45-µm and 0.22-µm filters. Quantification of HEV RNA in the virus inocula was determined according to the method described previously [17] and a final dilution containing 2.5×10^6^ copies per milliliter (mL) was immediately used for intravenous inoculation of animals.

### Immunization and challenge

Rabbits in both the vaccine and placebo groups were inoculated intramuscularly on week 0, 2 and 4 with a 30 µg dose of vaccine in 0.5 ml or 0.5 ml of PBS, respectively. The 6 rabbits in each group were further divided into two subgroups of 3 animals on week 6, and then challenged intravenously with 1 mL of either the genotype 4 swine HEV or the rabbit HEV inocula described above.

### Monitoring of rabbits following HEV challenge

Samples of serum and feces were collected prior to inoculation and weekly for 10 weeks after inoculation. Sera were tested immediately for levels of alanine aminotransferase (ALT) by a Hitachi Automatic Clinical Analyzer 7180 (Hitachi High-Technologies, Japan). Biochemical evidence of hepatitis was defined as a two-fold or greater increase in the post-inoculation/pre-inoculation ratio of ALT [18]. Anti-HEV antibodies in serum samples and HEV RNA in sera and feces were detected by ELISA and RT-nPCR as described elsewhere [8].

## Results

### Antibody responses of rabbits to the HEV 239 vaccine

**Figure 1:**
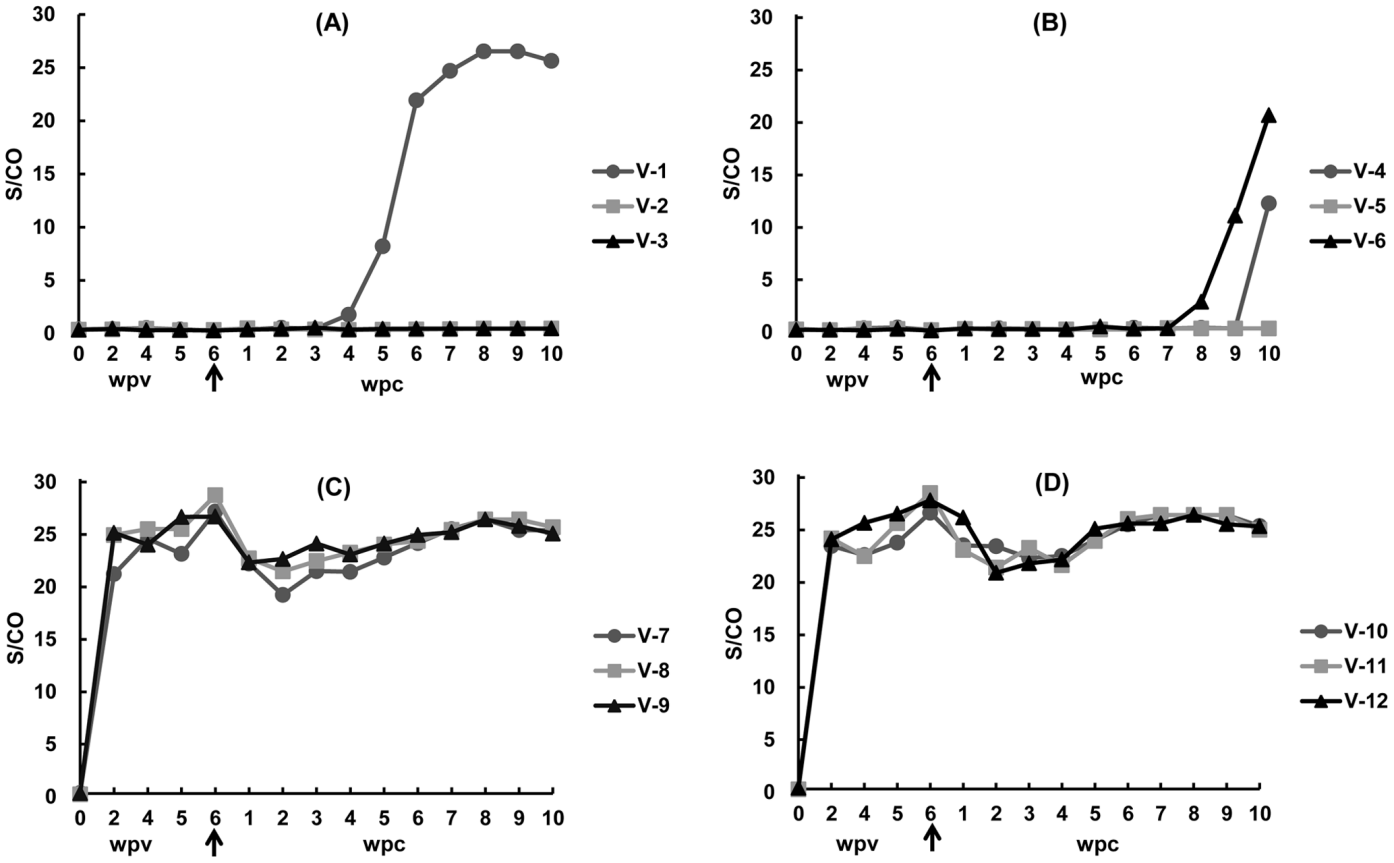
Time course of anti-HEV levels in rabbits vaccinated with HEV 239 vaccine and subsequently challenged with rabbit HEV and swine HEV at 6 weeks post-vaccination (wpv). (A & B) Rabbits inoculated with PBS buffer and challenged at 6 wpv with rabbit HEV and swine HEV of genotype 4, respectively. (C & D) Rabbits vaccinated with HEV 239 vaccine and challenged at 6 wpv with rabbit HEV and swine HEV of genotype 4, respectively. Arrow in the X-axis indicates the time of challenge at 6 wpv. Rabbits were vaccinated on week 0 and two booster doses on week 2 and week 4.

Each of the rabbits vaccinated with three 30 µg doses of the HEV 239 vaccine mounted an anti-HEV response while control animals inoculated with PBS remained negative for antibodies to HEV during the 6 week pre-challenge period. The first vaccine dose induced a strong anti-HEV antibody response in the vaccinated animals, with the mean signal-to-cutoff (S/CO) value reaching 23.5 at week 2. Following the two booster immunizations at the second and fourth week, the anti-HEV titer (S/CO) increased to 27.3 at the time of challenge (Figure 1).

### Protection against HEV infection in vaccinated rabbits following virus challenge

At week 6, each of the rabbits in groups A was challenged with 2.5×10^6^ copies of a rabbit HEV strain and those in group B with the same dose of a swine HEV strain of genotype 4.The course of the infection was monitored for 10 weeks after challenge by measuring serum ALT levels, viremia, fecal shedding and HEV antibody responses. Two of the control animals in group A inoculated with rabbit HEV showed significant elevations in serum ALT, as indicated by a peak ALT value that was equal to or greater than twice the geometric mean of the three immediate pre-challenge values (Table 1). Viremia and fecal shedding of virus was also detected in all three control rabbits in group A.HEV RNA was first detected in sera at 3 weeks after inoculation and was intermittently positive for a further 3–5 weeks, while virus excretion in stool was first detected 1 week post-challenge and continued until the end of monitoring at 10 weeks post challenge.

**Table 1:** HEV 239 vaccine protects rabbits against challenge with 10^4^ genomic dose of rabbit HEV or swine HEV of genotype 4*.

[Groups] vaccine	Rabbit ID#	Challenge inocula	Positive (+) or negative (−) HEV RNA detected in serum samples at indicated weeks post-challenge (wpc)											ALT (peak/pre)	Positive (+) or negative (−) HEV RNA detected in feces samples at indicated weeks post-challenge (wpc)										
			0	1	2	3	4	5	6	7	8	9	10		0	1	2	3	4	5	6	7	8	9	10
[A] 3× PBS	V-1	Rabbit HEV	**−**	**−**	**−**	**−**	**+**	**+**	**+**	**+**	**−**	**+**	**−**	2.6	–	**+**	**+**	**+**	**+**	**+**	**+**	**+**	**+**	**+**	**+**
	V-2		**−**	**−**	**−**	**−**	**−**	**+**	**+**	**+**	**−**	**−**	**−**	1.4	–	–	**+**	**+**	**+**	**+**	**+**	**+**	**+**	**+**	**+**
	V-3		**−**	**−**	**−**	**+**	**+**	**−**	**−**	**−**	**+**	**−**	**−**	2.2	–	–	**+**	**+**	**+**	**+**	**+**	**+**	**+**	**+**	**+**
[B] 3× PBS	V-4	Genotype 4 Swine HEV	**−**	**−**	**−**	**−**	**−**	**−**	**−**	**−**	**−**	**−**	**−**	2.5	–	–	–	–	–	–	–	**+**	**+**	**+**	**+**
	V-5		**−**	**−**	**−**	**−**	**−**	**−**	**−**	**−**	**−**	**−**	**−**	2.2	–	–	–	–	**+**	**+**	**+**	**+**	**+**	**+**	**+**
	V-6		**−**	**−**	**−**	**−**	**−**	**−**	**−**	**−**	**−**	**−**	**−**	2.0	–	–	–	–	**+**	**+**	**+**	**+**	**+**	**+**	**+**
[C] 3× 30 µg	V-7	Rabbit HEV	**−**	**−**	**−**	**−**	**−**	**−**	**−**	**−**	**−**	**−**	**−**	0.8	–	–	–	–	–	–	–	–	–	–	–
HEV 239	V-8		**−**	**−**	**−**	**−**	**−**	**−**	**−**	**−**	**−**	**−**	**−**	1.9	–	–	–	–	–	–	–	–	–	–	–
	V-9		**−**	**−**	**−**	**−**	**−**	**−**	**−**	**−**	**−**	**−**	**−**	1.3	–	–	–	–	–	–	–	–	–	–	–
[D] 3× 30 µg	V-10	Genotype 4 Swine HEV	**−**	**−**	**−**	**−**	**−**	**−**	**−**	**−**	**−**	**−**	**−**	1.0	–	**-**	–	–	–	–	–	–	–	–	–
HEV 239	V-11		**−**	**−**	**−**	**−**	**−**	**−**	**−**	**−**	**−**	**−**	**−**	1.2	–	–	–	–	–	–	–	–	–	–	–
	V-12		**−**	**−**	**−**	**−**	**−**	**−**	**−**	**−**	**−**	**−**	**−**	1.0	–	–	–	–	–	–	–	–	–	–	–

* HEV, hepatitis E virus; PBS, Phosphate Buffered Saline; ALT (peak/pre), peak/pre-infection ALT.

Although viremia was not detected in any of the control animals in group B inoculated with swine HEV genotype 4, the peak ALT values of each animal were greater than twice the pre-challenge values and all the control animals in this group excreted virus in the stool. Compared with that of rabbits in group A, fecal virus shedding was much delayed and viral RNA was first detected at 2 weeks post challenge and remained positive thereafter, up to the end of monitoring (Table 1).

At no time point after challenge, were there significant ALT elevations, viremia and fecal virus shedding detected in vaccinated rabbits in group C and D. After challenge, the levels of IgG anti-HEV antibodies maintained at similar levels with a slight decrease in titers in some rabbits towards the end of the study. A booster effect on IgG anti-HEV level after challenge was not evident, which is expected since the prior infection protected against the challenge.

## Discussion

Hepatitis E was historically thought to be a disease of developing countries associated with water-borne transmission. However, the recent recognition of the importance of chronic hepatitis E in immunosuppressed individuals [19]–[21] and the increasing number of zoonotically acquired cases from non-endemic industrialized countries has suggested that food-borne transmission appears to play a more important role in HEV infection in these regions. The recently licensed HEV 239 vaccine will be of great use in preventing of water-borne transmission during outbreaks in developing countries where the virus is endemic. However, prevention strategies for food-borne sporadic HEV infection in both developing and developed countries are more problematic since the target population and transmission routes are currently uncertain. Vaccination of the primary host for HEV seems a possible option [22], [23]. In the present study, we showed that the HEV 239 vaccine afforded complete protection for rabbits against challenge with rabbit HEV and swine HEV of genotype 4, suggesting the vaccine could be used to cope with zoonotic transmission of HEV.

The HEV 239 vaccine elicited excellent immunogenicity in rabbits, as indicated by the strong anti-HEV antibody response in the immunized group (Figure 1). This is consistent with previous findings in mice and rhesus monkeys [16]. Although the first dose had induced satisfactory immune responses with S/CO value reaching 23.5 at week 2, three 30µg doses of immunizations were carried out according to the instructions of the vaccine manufacture in this study. Consequently, additional studies should be performed to determine appropriate vaccine dosages and immunization times to make large-scale animal vaccinations much more cost effective. Besides, our results confirmed previous reports that rabbit model is able to serve as an alternative to the non-human primate models for HEV vaccine evaluation [24].

A growing body of evidence has revealed that rabbits constitute another potentially important reservoir of HEV and humans appear to be at risk of infection from rabbit HEV [8], [25]. The recent investigations of HEV prevalence in rabbits from China, USA and France indicated rabbit HEVs are widespread among various breeds of rabbits [26]–[32]. Moreover, the demonstration that rabbit HEV can experimentally infect cynomolgus macaques [8] and SPF pigs [33] together with the identification of a human HEV strain closely related to rabbit HEV [32] has reinforced the potential zoonotic risk of this virus. However, it remains unclear whether rabbit HEV is preventable by vaccination of HEV 239, which is based on a genotype 1 strain of HEV [16]. In the present study, the HEV 239 vaccine conferred cross-protection against subsequent infections from rabbit HEV and swine HEV of genotype 4. By contrast, all six unvaccinated rabbits developed hepatitis, with liver enzyme elevations, viremia, and fecal virus shedding following challenge. Previous studies have shown that the capsid protein of rabbit HEV cross-reacted with antibodies against human and swine HEV [33] and that rabbit and human HEV belong to a single serotype [34], which might explain why the genotype 1 vaccine is highly efficacious against both rabbit HEV and swine HEV infection. In the previous study with rhesus monkeys, the vaccine could afford complete protection against a challenge dose of 10^4^ genomes and partial protection against infection with 10^7^ genomes [16]. The titer of challenge virus in this study was only 2.5×10^6^ copies due to the limited amount of source material, and so additional studies will be needed to confirm that the 239 vaccine is able to protect rabbits against the higher challenge dose used in the earlier study.

In conclusion, the results from this study have demonstrated that the HEV 239 vaccine is highly immunogenic for rabbits and that it can completely protected these animals against rabbit HEV and genotype 4 HEV infections, suggesting the vaccine could serve as a candidate vaccine to manage animal sources of human HEV infection. These findings may facilitate the prevention of food-borne sporadic HEV infection in both developing and industrialized countries. Given that swine is the primary animal reservoir of HEV, future studies are warranted to evaluate the HEV 239 vaccine in the pig model.
